# Supplementary figures and images for: Africanized honeybee population (Apis mellifera L.) in Nicaragua: Forewing length and mitotype lineages
Source: PLoS One. 2022 Apr 25;17(4):e0267600. doi: 10.1371/journal.pone.0267600 (PMC9037913; doi:10.1371/journal.pone.0267600)

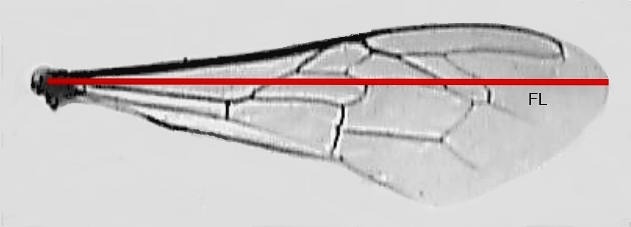

Supplement: S1 Fig — (TIFF) [file pone.0267600.s001.tiff]
